# Supplementary material for: Universal vibrational anharmonicity in carbyne-like materials
Source: Nat Commun. 2025 May 10;16:4360. doi: 10.1038/s41467-025-59555-y (PMC12065905; doi:10.1038/s41467-025-59555-y)
Supplement: Supplementary file 1 — Supplementary Information [file 41467_2025_59555_MOESM1_ESM.pdf]

Supplementary Information for:
Universal Vibrational Anharmonicity in Carbyne-like Materials

Johannes M. A. Lechner,[1] Pietro Marabotti,[1] Lei Shi,[2] Thomas Pichler,[3] Carlo Spartaco Casari,[4] and Sebastian Heeg*,[1]

[1]*Institut für Physik, Humboldt-Universität zu Berlin, Berlin, Germany*

[2]*School of Materials Science and Engineering, Sun Yat-sen University, Guangzhou, China*

[3]*Fakultät für Physik, Universität Wien, Wien, Austria*

[4]*Dipartimento di Energia, Politecnico di Milano, Milano, Italy*

E-mail: sebastian.heeg@physik.hu-berlin.de

Contents

Suppl. Note 1: Raman spectra of confined carbyne chains and selection criteria **3**

Suppl. Note 2: Evaluation of anharmonicity of BLA oscillation using VPT2 **5**

Suppl. Note 3: Limitations of VPT4 for anharmonicity analysis **8**

Suppl. Note 4: Experimental data and anharmonic parameters **11**

Suppl. Note 5: Total anharmonic redshifts and look-up table **12**

Supplementary References **13**

Suppl. Note 1: Raman spectra of confined carbyne chains and selection criteria

Typical Raman spectra of the confined carbyne chains measured for this work are presented in Suppl. Fig. 1. Since the spectrometer used does not capture the whole spectral bandwith from the C mode to the 4C mode in a single spectrum, two spectra are recorded for each location where the 4C mode could be detected. The first spectrum, 1(a) and (c), captures

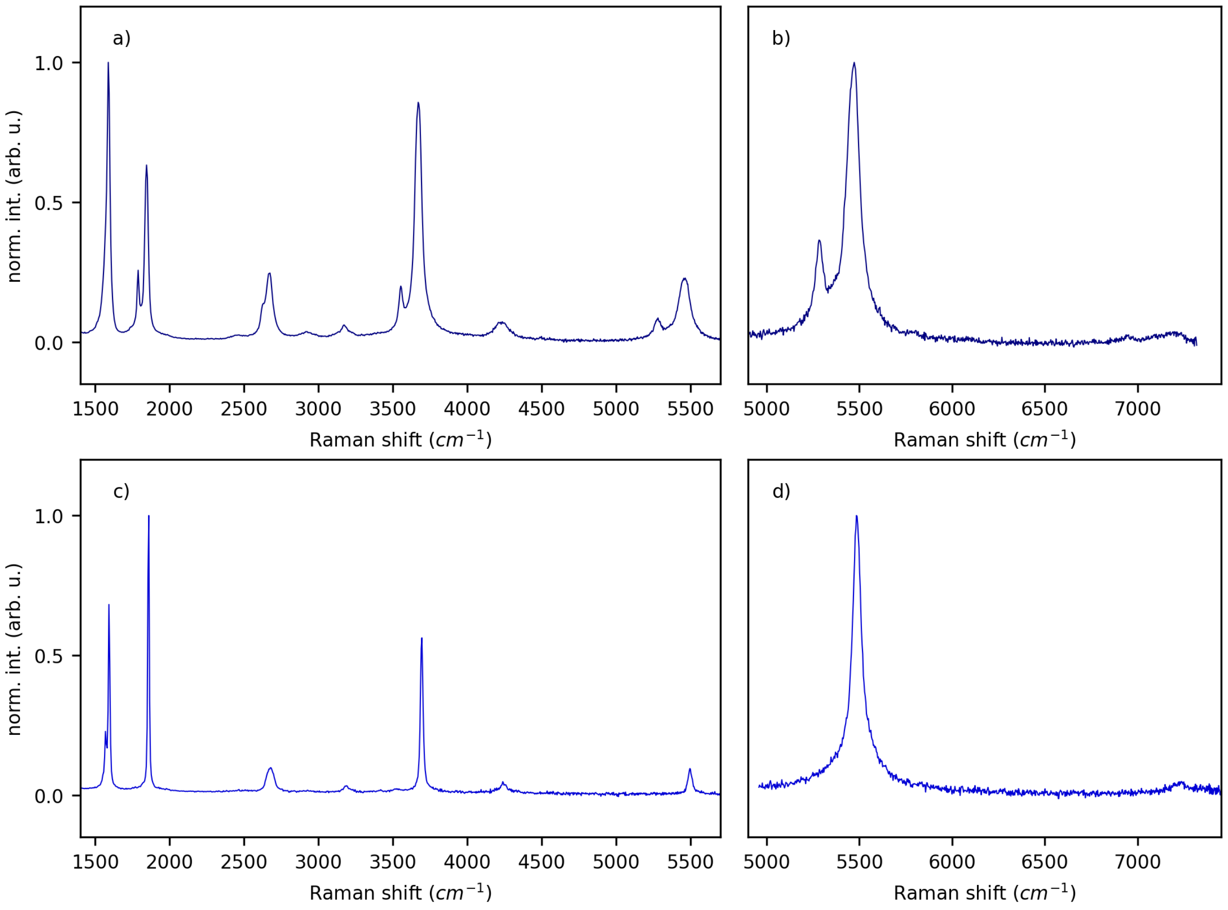

Supplementary Figure 1: **Raman spectra of confined carbyne chains at two separate locations.** (a) and (b) show spectra of a location where two confined carbyne chains are probed within the laser spot. Their C mode and overtones can be spectrally resolved unambiguously. (c) and (d) show spectra of a location with a single isolated confined carbyne chain. (a) and (c) show the C, 2C and 3C modes, while (b) and (d) show the 3C and 4C modes of the corresponding chain(s). A separately measured background has been subtracted from all spectra.

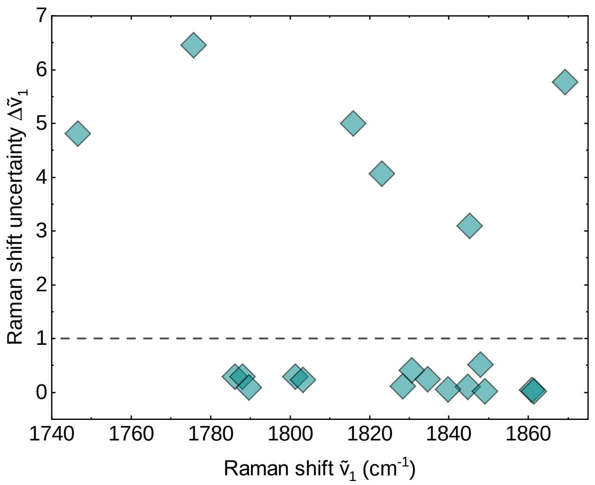

Supplementary Figure 2: **Positional fitting uncertainty** $\Delta\widetilde{\nu}_1$ of the Lorentzian functions used to fit the C mode of confined carbyne Raman spectra. If the positional uncertainty $\Delta\widetilde{\nu}_1 > 1$ cm^{-1}, the corresponding peak is excluded from our analysis.

the C, 2C and 3C modes, while the second spectrum, 1(b) and (d), captures the 3C and 4C mode.

Other than the C mode and its overtones, several more peaks appear in the measured spectral range, which originates from the host nanotubes. The well known G mode of CNTs can be found just below 1600 cm^{-1}, while the 2D mode appears at about 2700 cm^{-1}.[1] At roughly 3200 cm^{-1} the second-order of the G mode also appears. The G + 2D combination mode is visible at 4300 cm^{-1}. In some spectra a small peak at 2900 cm^{-1} appears, which has been assigned to either a D + D' combination mode or a double-resonant overtone of a $q \neq 0$ LO phonon.[2,3]

We determine the Raman shifts of the confined carbyne Raman peaks with Lorentzian fit functions. In order to quantify the anharmonicity in confined carbyne with high precision, only chains with well-resolved peaks with negligible fitting error are included in our analysis. We consider a confined carbyne chain well-resolved if the fitting uncertainty corresponding to the spectral position of the Lorentzian fit function of its C mode is smaller than 1 cm^{-1}. This parameter is plotted in Suppl. Fig. 2 for the functions used to fit the C mode of all

chains. This value is somewhat arbitrary but it is comparable to the resolution of scientific grade Raman spectrometers. Further, this value separates a group of chains with C modes that gave very little fitting uncertainty from chains whose C modes can only be fitted well with larger errors of several cm^{-1} .

Additionally, spectra that show major contributions from more than 2 chains are excluded to decrease fitting uncertainty and prevent erroneous attribution of overtones to the wrong confined carbyne chain. This is considered to be the case if the integrated area of the third-largest Lorentzian fit function used for a certain peak is at least 10% as large as the integrated area of the second-largest Lorentzian fit function.

Based on the selection criteria described above, the Raman shift of the C mode and overtones of the confined carbyne chains used for this study are reported in Suppl. Table 1 together with the corresponding values of carbon atomic wires from Ref. 4.

Suppl. Note 2: Evaluation of anharmonicity of BLA oscillation using VPT2

In the framework of second-order vibrational perturbation theory (VTP2),[5] anharmonic vibrational levels can be derived by introducing an anharmonic correction matrix (χ). This method describes the perturbed potential energy curve as a fourth-degree polynomial. In the single-mode approximation and considering no mode mixing (as confirmed by our experimental data, see the Results section in the main text), valid for the ideal carbyne system and confined carbyne, the harmonic expression of the potential energy is corrected by adding terms proportional to q^3 and q^4, where q is the vibrational normal coordinate. The general expression for the vibrational level of quantum number n is expressed by Eq. 1 in the main text and reported here for convenience:

$$\frac{E_n}{hc} = \varepsilon_0 + \tilde{\nu}_{harm} n - \tilde{\nu}_{harm} \chi \left(n^2 + n\right) , \qquad \text{(S. 1)}$$

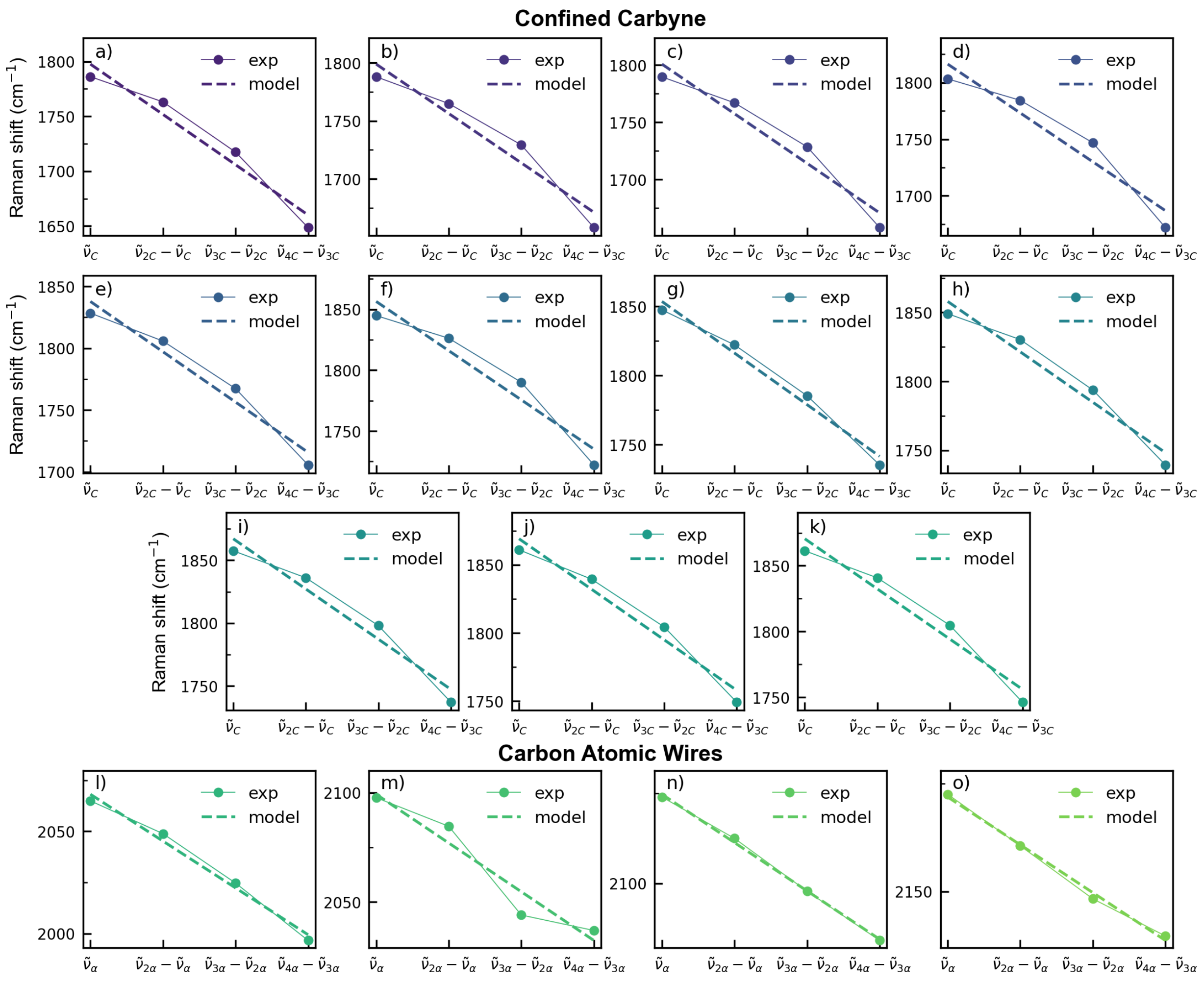

Supplementary Figure 3: **Modeling of anharmonic redshifts according to VPT2** a-k) Frequency spacing between two subsequent vibrational quanta of the C mode (colored circles, ($\tilde{\nu}_{(n+1)C} - \tilde{\nu}_{nC}$, with n=1,2,3,...) and results of the linear fit procedure (dotted lines, see Eq. S. 2) for confined carbyne. l-o) Frequency spacing between two subsequent vibrational quanta of the α mode (colored circles, ($\tilde{\nu}_{(n+1)\alpha} - \tilde{\nu}_{n\alpha}$, with n=1,2,3,...) and results of the linear fit procedure (dotted lines, see Eq. S. 2) for carbon atomic wires from Ref. 4.

where ε_0 is the zero-point vibrational energy, $\tilde{\nu}_{harm}$ is the harmonic frequency (in cm^{-1}) of the vibrational mode, and χ is the nondimensional anharmonic parameter. For the difference between the $(n+1)^{th}$ and n^{th} levels, we obtain

$$\frac{E_{n+1} - E_n}{hc} = \tilde{\nu}_{harm}\,(1 - 2\chi) - (2\chi\tilde{\nu}_{harm})\,n = K - Sn. \qquad \text{(S. 2)}$$

This relation relies exclusively on parameters that can be determined experimentally by

Raman spectroscopy and does not require assuming values that cannot be determined, such as, i.e., the zero-point vibrational energy. Furthermore, we can use Eq. S. 2 to model our experimental data with a linear regression. From the intercept (K) and the slope (S), we can calculate $\tilde{\nu}_{harm}$ and χ as

$$\begin{aligned} \tilde{\nu}_{harm} &= K + S \\ \chi &= \frac{1}{2\,(1 + K/S)}. \end{aligned} \tag{S. 3}$$

Among the 16 confined carbyne chains available, we selected only 11 for which we can detect up to the third overtone (4C mode), listed in Suppl. Table 1. In this work, we compared confined carbyne chains to short carbon atomic wires, whose Raman spectra and the frequencies of their corresponding α mode and overtones are displayed in Ref. 4. Suppl. Fig. 3 illustrates the experimental frequency spacings between subsequent vibrational levels in confined carbyne chains and short carbon atomics wires, along with the results of linear regression, while the values of $\tilde{\nu}_{harm}$ and χ are reported in Suppl. Table 1.

VPT2 offers a reliable and straightforward method for quantifying anharmonicity. By using only two fitting parameters, χ and $\tilde{\nu}_{harm}$, VPT2 avoids the risk of overfitting, making it well-suited for our experimental dataset comprised of 4 data points for each chain. As a result, χ shows reduced fluctuations and a clear correlation with the C/ECC mode frequency (see Fig. 4 and Suppl. Fig. 4e), while VPT2's $\tilde{\nu}_{harm}$ closely follows a linear trend with C/ECC mode frequency as would be expected (Suppl. Fig. 4g). Finally, the residuals from VPT2 show little variance (Suppl. Fig. 4h), reinforcing the reliability and robustness of the VPT2 model against experimental and systematic fitting uncertainties. Note that VPT2 does not fully capture the super-linear decrease in frequency spacing with mode order, Suppl. Fig. 3. This leads to systematic fitting errors that are reflected in the rather large uncertainties for χ in Fig. 4 of the main paper. The nature of these uncertainties, however, explains their negligible effect on our analysis and confirms that VPT2 captures the essential physics behind the our experimental data within reasonable accuracy. For these reasons, we consider VPT2 the appropriate method to apply to this particular dataset.

Suppl. Note 3: Limitations of VPT4 for anharmonicity analysis

As shown in Figure 3, a linear fit based on VPT2 does not fully describe the decrease in vibrational energy spacing of confined carbyne chains. To increase the degree of the polynomial fit (*i.e.*, , to a quadratic function), we have to introduce an additional anharmonic parameter. This implies moving from VPT2 to VPT4 where a sixth-degree polynomial (q^5 and q^6) has been used to model the perturbed potential energy curve.[6] According to Gong *et al.*,[6] two anharmonic correction matrices (χ and ψ) are introduced to express the vibrational energy levels. Using the same approximations employed for VPT2 in Section Suppl. Note 2:, we obtain

$$\frac{E_n}{hc} = \varepsilon_0 + \tilde{\nu}_{harm} n + \tilde{\nu}_{harm}\chi\left(n^2+n\right) + \tilde{\nu}_{harm}\psi\left(n^3+\frac{3}{2}n^2+\frac{3}{2}n\right). \tag{S. 4}$$

The different sign in front of the linear term depending on χ compared to VPT2 implies that in VPT4 the first-order anharmonic correction may bring either a positive or negative contribution to the harmonic frequency. Here, the difference between the $(n+1)^{th}$ and n^{th} levels becomes

$$\frac{E_{n+1}-E_n}{hc} = \tilde{\nu}_{harm}\left(1+2\chi+\frac{13}{4}\psi\right) + \left(2\chi+6\psi\right)\tilde{\nu}_{harm} n + \left(2\psi\right)\tilde{\nu}_{harm} n^2 = K + Sn + Rn^2. \tag{S. 5}$$

Using a quadratic curve to model our experimental data, from the set of fitting parameters (K, S, and R), we can extract $\tilde{\nu}_{harm}$, χ, and ψ:

$$\begin{aligned} \tilde{\nu}_{harm} &= K - S + \frac{11}{12}R \\ \chi &= \frac{12R-6S}{11R-12S+12K} \\ \psi &= \frac{4R}{11R-12S+12K} \end{aligned} \tag{S. 6}$$

Figure 4a-d compares illustrative results of the regression of individual chains using VPT2

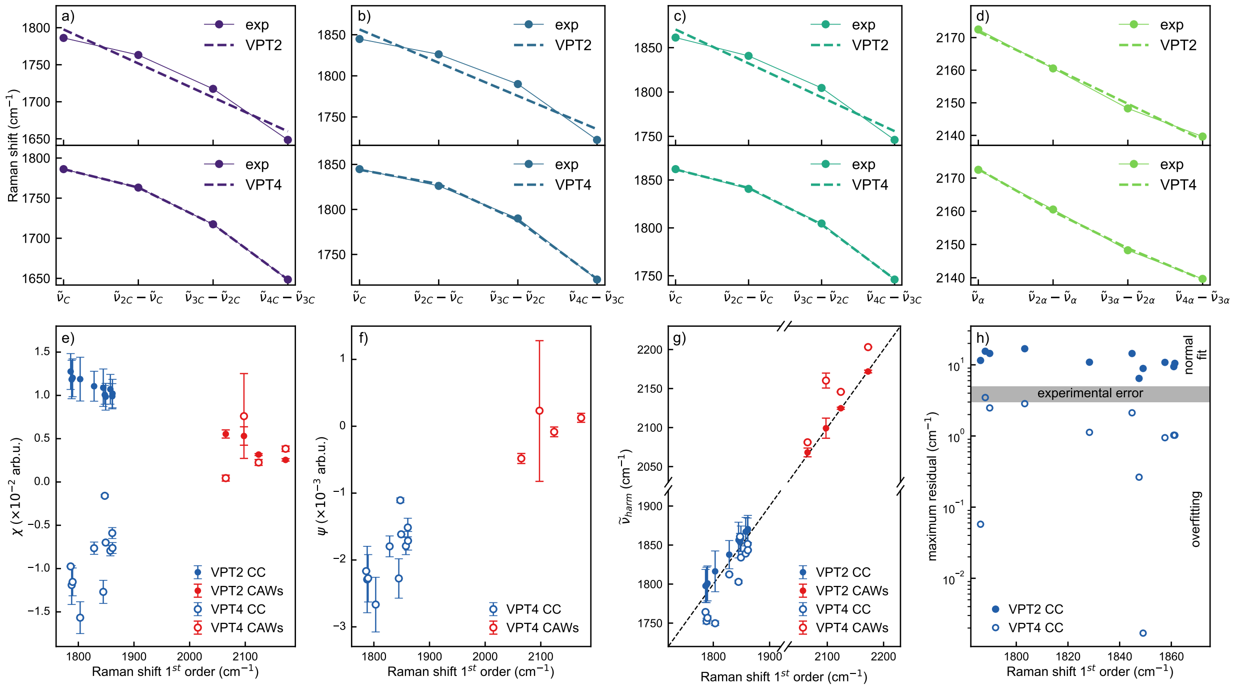

Supplementary Figure 4: **VPT4 and VPT2 comparison** a)-d) Exemplary fits of individual chains with the VPT2 (upper panels) and VPT4 (lower panels) models. e) χ, f) ψ and g) $\tilde{\nu}_{harm}$ fit parameters of confined carbyne chains (blue) and carbon atomic wires (red) as a function of the Raman shift of their corresponding first-order mode according to VPT2 (filled circles) and VPT4 (open circles). The errors derive from the fit of the vibrational energy spacing of confined carbyne and carbon atomic wires using VPT2 and VPT4 (see Eqs. S. 5 and S. 6). h) Maximum residuals of VPT2 (filled circles) and VPT4 (empty circles) fits as a function of Raman shift of their corresponding first-order mode.

(upper panels, see Suppl. Fig. 3 for the complete dataset) to the fitting using VPT4 (lower panels). At first glance, VPT4 seems to align well with the experimental data. However, a closer inspection reveals significant limitations of VPT4 when applied to our dataset. Due to the large energy separation of overtones (230 meV), a maximum of 4 data points per chain can be measured, which are fitted with 3 variables within the VPT4 approximation. This makes the fitting statistically underdetermined and leads to severe overfitting. Overfitting is evident in the large variation in all VPT4 fitting parameters χ, ψ, and $\tilde{\nu}_{harm}$, shown in panels e) to g) of Suppl. Fig. 4. If VPT4 accurately captured the anharmonicity across all chains, the parameters χ, ψ, and $\tilde{\nu}_{harm}$ would exhibit homogenous continuous trends. Instead, the observed fluctuations indicate that the model is unstable and overly sensitive to

small variations in the dataset due to experimental error, which is a hallmark of overfitting.

Finally, the overfitting issue clearly appears when reviewing the fit residuals (observed value - predicted value) from the VPT4 fits of the anharmonic redshifts shown in Suppl. Fig. 4h. The residuals are all smaller than the experimental error of around 3-5 cm^{-1}, with some being much smaller (up to three orders of magnitude) to a degree that would be very unlikely in a sufficiently stable model. This renders VPT4 entirely unsuitable to fit our data.

Suppl. Note 4: Experimental data and anharmonic parameters

Supplementary Table 1: Experimental Raman modes of confined carbyne (C mode) and carbon atomic wires (ECC or α mode), ideal harmonic frequency ($\tilde{\nu}_{harm}$), and anharmonic parameter (χ) for each carbyne-like system, calculated using Eq. S. 3.

**Confined Carbyne**					
Raman shift				VPT2	
$\tilde{\nu}_{1C}$ $[cm^{-1}]$	$\tilde{\nu}_{2C}$ $[cm^{-1}]$	$\tilde{\nu}_{3C}$ $[cm^{-1}]$	$\tilde{\nu}_{4C}$ $[cm^{-1}]$	$\tilde{\nu}_{harm}$ $[cm^{-1}]$	χ
1786	3549	5267	6915	1798	0.0128
1788	3553	5282	6940	1799	0.0118
1790	3557	5285	6944	1801	0.012
1803	3588	5334	7006	1816	0.0119
1828	3634	5401	7107	1838	0.0111
1845	3671	5461	7183	1856	0.0109
1848	3670	5455	7191	1854	0.0101
1849	3679	5473	7212	1858	0.0098
1858	3694	5492	7229	1867	0.0107
1861	3700	5505	7254	1869	0.0099
1861	3702	5507	7253	1870	0.0102
1801	3581	5322	-	-	-
1831	3638	5412	-	-	-
1835	3651	5430	-	-	-
1840	3654	5431	-	-	-
1848	3681	5489	-	-	-
**Carbon atomic wires**					
Raman shift				VPT2	
$\tilde{\nu}_{1\alpha}$ $[cm^{-1}]$	$\tilde{\nu}_{2\alpha}$ $[cm^{-1}]$	$\tilde{\nu}_{3\alpha}$ $[cm^{-1}]$	$\tilde{\nu}_{4\alpha}$ $[cm^{-1}]$	$\tilde{\nu}_{harm}$ $[cm^{-1}]$	χ
2065	4114	6139	8135	2068	0.0055
2098	4182	6226	8263	2099	0.0053
2124	4237	6334	8418	2125	0.0032
2172	4333	6481	8621	2172	0.0025

Suppl. Note 5: Total anharmonic redshifts and look-up table

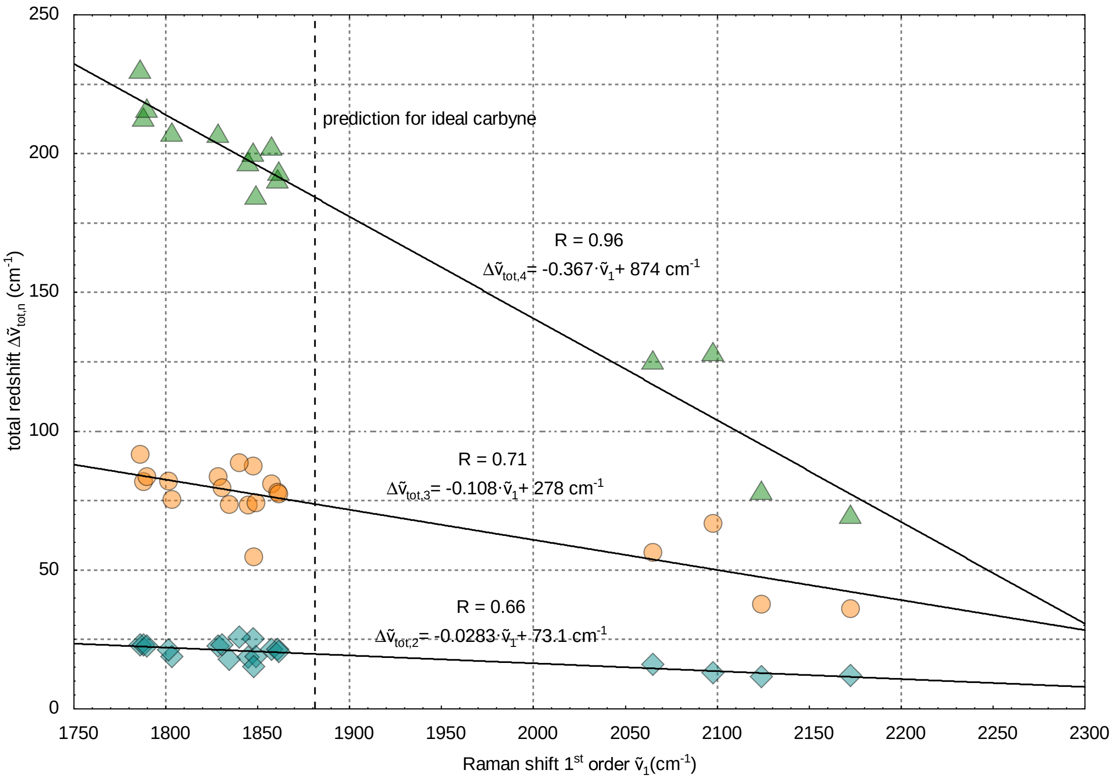

Supplementary Figure 5: **Total anharmonic redshift** $\Delta\widetilde{\nu}_{tot,n} = n \cdot \widetilde{\nu}_1 - \widetilde{\nu}_n$ as a function of the fundamental BLA oscillation mode frequency for confined carbyne and carbon atomic wires up to the 4th-order. Linear trends and their equations are included.

In Suppl. Fig. 5 we provide the total anharmonic redshift of the overtones of confined carbyne and carbon atomic wires relative to the corresponding multiple of fundamental BLA oscillation mode (C mode/ECC mode). The increase in the anharmonic overtone redshift with decreasing BLA oscillation follows linear trends and their parameters are provided in the Figure. The total anharmonic redshifts expected for ideal carbyne are indicated by the dashed line.[7] We expect alternative carbyne-like materials to follow the same relations. Figure 5 may hence serve as a look-up table to confirm that a material is carbyne-like based on its BLA oscillation and overtone frequencies.

Supplementary References

(1) Reich, S.; Thomsen, C.; Maultzsch, J. *Carbon nanotubes: basic concepts and physical properties*; John Wiley & Sons, 2008.

(2) Fantini, C.; Pimenta, M. A.; Strano, M. S. Two-Phonon Combination Raman Modes in Covalently Functionalized Single-Wall Carbon Nanotubes. *The Journal of Physical Chemistry C* **2008**, *112*, 13150–13155.

(3) Dresselhaus, M.; Dresselhaus, G.; Saito, R.; Jorio, A. Raman spectroscopy of carbon nanotubes. *Physics Reports* **2005**, *409*, 47–99.

(4) Marabotti, P.; Tommasini, M.; Castiglioni, C.; Peggiani, S.; Serafini, P.; Rossi, B.; Bassi, A. L.; Russo, V.; Casari, C. S. Synchrotron-based UV resonance Raman spectroscopy probes size confinement, termination effects, and anharmonicity of carbon atomic wires. *Carbon* **2024**, *216*, 118503.

(5) Mendolicchio, M.; Bloino, J.; Barone, V. Perturb-then-diagonalize vibrational engine exploiting curvilinear internal coordinates. *J. Chem. Theor. Comput.* **2022**, *18*, 7603–7619.

(6) Gong, J. Z.; Matthews, D. A.; Changala, P. B.; Stanton, J. F. Fourth-order vibrational perturbation theory with the Watson Hamiltonian: Report of working equations and preliminary results. *J. Chem. Phys.* **2018**, *149*.

(7) Arora, A.; Baksi, S. D.; Weisbach, N.; Amini, H.; Bhuvanesh, N.; Gladysz, J. A. Monodisperse Molecular Models for the sp Carbon Allotrope Carbyne; Syntheses, Structures, and Properties of Diplatinum Polyynediyl Complexes with PtC20Pt to PtC52Pt Linkages. *ACS Central Science* **2023**, *9*, 2225–2240.
